# Nutritional Values and Bio-Functional Properties of Fungal Proteins: Applications in Foods as a Sustainable Source

**DOI:** 10.3390/foods12244388

**Published:** 2023-12-06

**Authors:** Ku Li, Kaina Qiao, Jian Xiong, Hui Guo, Yuyu Zhang

**Affiliations:** 1 Hubei Provincial Key Laboratory of Yeast Function, Angel Yeast Co., Ltd., 168 Chengdu Road, Yichang 443003, China; 2 Key Laboratory of Flavor Science of China General Chamber of Commerce, Beijing Technology and Business University, Beijing 100048, China; 3 Key Laboratory of Geriatric Nutrition and Health, Beijing Technology and Business University, Ministry of Education, Beijing 100048, China

**Keywords:** fungus, fungal proteins, nutritional values, bio-functional properties, applications

## Abstract

From the preparation of bread, cheese, beer, and condiments to vegetarian meat products, fungi play a leading role in the food fermentation industry. With the shortage of global protein resources and the decrease in cultivated land, fungal protein has received much attention for its sustainability. Fungi are high in protein, rich in amino acids, low in fat, and almost cholesterol-free. These properties mean they could be used as a promising supplement for animal and plant proteins. The selection of strains and the fermentation process dominate the flavor and quality of fungal-protein-based products. In terms of function, fungal proteins exhibit better digestive properties, can regulate blood lipid and cholesterol levels, improve immunity, and promote gut health. However, consumer acceptance of fungal proteins is low due to their flavor and safety. Thus, this review puts forward prospects in terms of these issues.

## 1. Introduction

Proteins are the material basis of almost all life on Earth. The birth, existence, and death of living things are all related to proteins. Specifically, proteins are essential for cell renewal and repair; they are the physiological basis of immunity, the regulators of physiological functions (such as enzymes and hormones), and a source of energy for sustaining vital activities [1]. The main source of protein for human is animal proteins (meat, poultry, eggs, or seafood), followed by plant proteins (nuts, seeds, beans, or soy products). These foods are also crucial sources of trace elements, amino acids, choline, and fatty acids for the human body. The World Health Organization (WHO) recommends that adults consume 0.75 g (each kilogram of weight) protein per day, and the recommended protein intake varies in different countries. For example, the Dietary Guidelines for Americans 2020–2025 recommend that adults consume 2.3–3.1 g/day (per kilogram of weight) of protein [2]. The Chinese Dietary Reference Intakes recommend a protein intake for adults of 0.8–1.0 g/day (per kilogram of weight) [3]. The European Society for Clinical and Economic Aspects of Osteoporosis and Osteoarthritis (ESCEO) recommends the optimal protein intake from foods of 1.0–1.2 g/day (each kilogram of weight) [4]. By 2050, the global population is estimated to grow to 10 billion, and, as a result, the requirements for food will increase by 50%, while the human demand for animal-derived protein will nearly triple [5]. According to the data from the Food and Agriculture Organization of the United Nations, meat consumption will reach 455 million tons by 2050 around the world, and greenhouse gas released from agricultural land will account for more than 30% of human-made emissions [6]. However, the existing protein acquisition mostly relies on aquaculture, land, and water, which cannot cope with the huge pressure resulting from continuous population growth and the improvement of living standards [6]. It is essential to search for sustainable alternative sources of dietary proteins. Therefore, the manufacture of microbial proteins has attracted extensive interest.

Fungi, as heterotrophic eukaryotic micro-organisms, obtain food mainly through absorbing soluble organic substances on the cell surface [7]. Fungi occupy a large part of the microbial family and possess the ability to grow easily, these properties making large-scale production possible. Fungi are widely used in industrial production, such as for vitamins, enzymes, antibiotics, pigments, fatty acids, amino acids, polysaccharides, polyols, and glycolipids [8]. In addition, the secondary metabolites of fungi are crucial to maintaining human health and treating diseases, and possess huge economic benefits. For example, fungi play an important role in the production of certain drugs, such as penicillin derived from *Penicillium*, lovastatin extracted from *Aspergillus terreus*, alkaloids and polyketide compounds isolated from *Cladosporium*, etc., and they represent a resource to be further exploited with great potential for industrial production [9]. Among fungi, mushrooms and mycelia can provide dietary protein, lipids and fatty acids, vitamins, and cellulose, and improve the sensory properties and flavor of processed foods as a meat substitute [10]. Since different strains can produce different specialized metabolites, they are often key components of nutritional supplements [11,12]. Fungi are widely used in food because of their ability to break down organic substances and produce attractive flavors, colors, and textures. For example, *Aspergillus oryzae* and *Aspergillus sojae* are used in the production of Jiuqu and soy sauce [13], *Aspergillus niger* is used in deep fermentation to yield citric acid and enzymes [14], and yeast is used to make bread and wine. The contribution of fungi to the global market through fungal products and services is estimated at USD 54,574.53 million, and the industrial use of fungi in food and beverages is valued at 2141.18 USD billion [15]. Fungi also produce some secondary metabolites with important applications in human life, such as penicillin. However, the secondary metabolites produced by fungi can also be harmful to humans. Mycotoxins are a group of toxic secondary metabolites produced by some fungi under suitable conditions, and are also the most common type of food contaminants, exhibiting carcinogenicity, teratogenicity, neurotoxicity, and other characteristics [16,17]. Aflatoxins are the toxic secondary metabolites secreted by *Aspergillus flavus* and *Aspergillus parasiticus* in contaminated food. Strains of *Fusarium* produce zearalenone and fumonisins; moreover, fungi such as *Aspergillus* and *Penicillium* produce ochratoxins, which are all harmful to human health [18,19,20,21].

By 2050, substituting 20% of the per capita intake of ruminant meat with microprotein would compensate for the required increase in the global grazing area and cut deforestation by 56%, net CO_2_ emissions by 56%, and methane emissions by 11%. Replacing 50% or 80% of the per capita intake of ruminant meat with microbial protein could reduce deforestation by 82% or 93%, net CO_2_ emissions by 83% or 87%, and methane emissions by 26% or 39%, respectively [22]. Therefore, the large-scale and safe production of microbial proteins, especially fungal proteins, is extremely important. Fungal-protein-based products can maintain their mycelial structure during food processing, because their filamentous cells have a similar texture to the protein fibrils in meat tissues, thus making fungal protein products a good meat substitute [23]. Moreover, fungal-protein-based foods have evolved from mushroom products to other processed foods such as beverages, non-dairy cheese, milk substitutes, and so on. As well as being edible, fungal proteins have a series of health benefits for humans, including improving blood lipids, increasing dietary fiber intake, promoting satiety, and muscle fiber synthesis [24]. Thus, this review aims to summarize the nutritional values and bio-functional properties of fungal proteins, in order to provide some guidance for the production of fungal-protein-based foods. 

**Figure 1:**
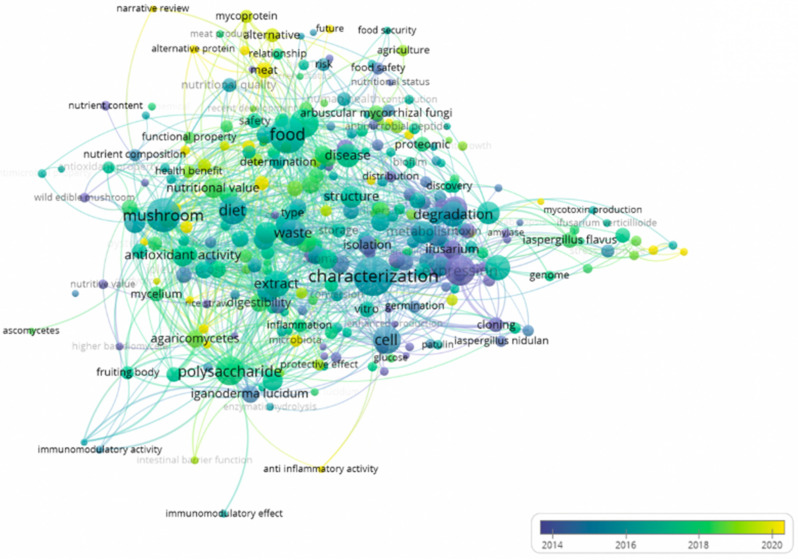
Network visualization of the titles and abstracts in the selected 10,000 studies related to topics regarding the nutrition and function of fungal proteins from 2000 to 2024.

The study trends and topics with respect to fungal proteins (nutrition, function, health, safety, and sustainability) in the selected 10,000 studies, which were found in Web of Science during the period 2000–2024, were summarized using VOSviewer software (version 1.6.18, Leiden University, Leiden, The Netherlands). The minimum frequency of keyword occurrence was set as 10, and a clustering co-occurrence map was drawn. The results of the bibliometrics and visualization analysis were used to objectively list the current research status, hotspots, development background, and expected research topics in this direction. Each circular node represents a keyword, a line represents the co-occurrence relationship between the two keywords, and the color of the node represents different clusters. The results of the network visualization are presented in Figure 1. In the past five years, the research topics surrounding fungal protein have related to nutritional value, health benefits, alternatives, safety in food application, and functional properties including antioxidant and anti-inflammatory properties.

## 2. Properties of Fungal Proteins

### 2.1. Varieties of Fungus

More than 120,000 species of fungi have been identified, of which over 6000 can form large fruiting bodies or sclerotium tissue. However, only about 2000 species of fungi can be used as food. Many fungi are single-celled and settle on the surface of a particle, producing extensions that then pierce the solid particle, extracting nutrients from inside the cells [7]. The terms used to classify fungi are the same as for other organisms; namely, kingdom, division, subphylum, class, order, family, genus, and species. According to the recognized G.C. Ainsworth fungal classification system, the total number of fungi is about 100,000 to 250,000. These can be categorized into seven divisions [25,26] including *Chytridiomycota*, *Blastocladiomycota*, *Neocallimastigomycota*, *Glomeromycota*, *Zygomycota*, *Ascomycota*, and *Basidiomycota*. The common feature of *Zygomycotina* is the production of zygospores by sexual reproduction, whereas asexual reproduction produces sporangia and sporospores, such as *Mucor*, *Rhizopus*, and *Rhizopus nigrocans*. The mycelium is developed without gaps and is luxuriant [27]. *Ascomycotina* is the largest subfamily of fungi, with about 15,000 species. Except for yeast, which is a single-celled organism, most vegetative bodies have developed mycelia. Mycelia have transverse septa, producing sexual spores (ascospores). Among them, *Neurospora* and *Saccharomyces* are the most common [28]. Yeast is by far the most widely used fungus, and rich in active sugars, peptides, nucleotides, amino acids, vitamins, and other natural ingredients, and is widely used in biomedicine, health food, and cosmetics. Yeast comprise nearly 1500 species and are commonly used in industry, mainly including *Saccharomyces cerevisiae*, *Pichia pastoris*, *Saccharomyces boulardii*, *Rhodotorula glutinis*, *Candida*, etc. [29]. *Basidiomycotina* has a vegetative body, which comprises a single cell or septate hypha and produces sexual spores [7]. Edible fungi (mushrooms) are mostly *Basidiomycetes* and can be commercially produced on organic compound fertilizers. *Mold* is a kind of fungus with a more developed mycelium and no large fruiting body. Macrofungi include *Mdulis*, Mushrooms, *Lentinus edodes*, *Tremella*, *Ganoderma lucidum*, *Hericium erinaceus*, *Dictyophora indusiata*, and so on. The main fungi that are being used to produce fungal proteins include mushrooms (*Basidiomycetes*) and *Fusarium venenatum*, as well as *Ascomycetes* and *Zygomycetes* (*Aspergillus*, *Rhyzopus*, and *Neurospora*), using solid-state fermentation to produce fungal proteins.

### 2.2. The Composition of Fungal Proteins

Fungi contain 20–30%-dry-matter crude protein, which contains all the essential amino acids (EAAs) and can meet the nutritional needs of humans and animals for proteins. Fungi contain a large amount of nucleic acid (8–25%), most of which is ribonucleic acid (RNA). In order to reduce the damage on human health, fungal proteins must be processed in a certain way to reduce the content of RNA (to less than 2%), and decrease the risk of gout. According to the European Commission criteria, fungal protein can be classified as “high fiber” as it provides at least 6 g fiber per 100 g [30]. Fungal proteins are low in total fat and saturated fat and have inappreciable cholesterol contents [31]. The content of saturated fatty acids is low, but the content of polyunsaturated fatty acids is high [6]. The composition of fat in fungal proteins is closer to that of plant fats, and the ratio of unsaturated to saturated fatty acids is about 4:1. Among them, the essential fatty acids linoleic acid and linolenic acid cannot be synthesized in the human body and have a certain nutritional value and physiological activity. Moreover, fungal proteins provide a series of micronutrients, including vitamin B12, riboflavin, folate, phosphorus, choline, potassium, zinc, and manganese [32]. The relevant information is presented in Table 1.

### 2.3. Extraction of Fungal Proteins

**Figure 2:**
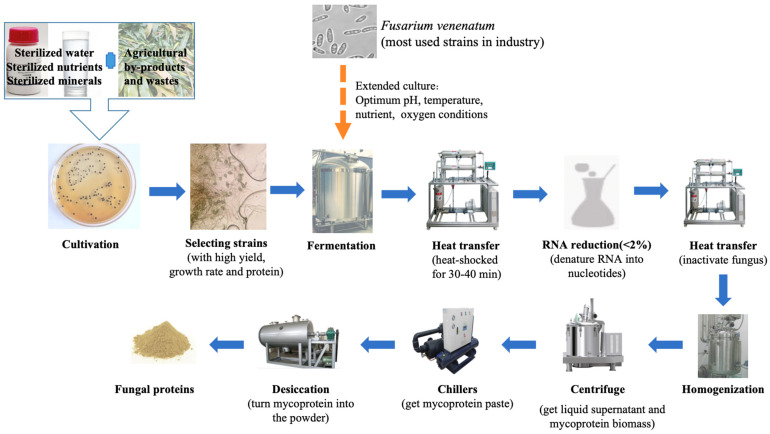
The production process of fungal proteins.

Fungal proteins can be produced in large quantities using industrial fermenters. Detailed steps are shown in Figure 2. First, strains are cultured in sterilized water, nutrient, and mineral combined substrates, such as agricultural by-products or wastes. Then, strains with a high yield, growth rate, and protein content are selected for further fermentation. Fungal solid-state fermentation has an impact on its protein contents, amino acid composition, protein bioaccessibility, the flavor of end products, and other aspects. Solid-state fermentation can promote the transition of fungi into enzymes and edible ingredients. Studies have confirmed that fungal solid-state fermentation can improve nutritional status and bioaccessibility [33]. In general, *Fusarium venenatum* is the most commonly used strain for industrial fungal protein production, because it has a variety of nutritional values, such as a protein content of more than 41%, dry biomass fat content of 13%, and dietary fiber content of 25% [34,35]. During the fermentation process, more glucose, oxygen, and ammonia are added to the fermenter to help the fungus continue to grow and produce further cultures. Optimal pH, temperature, nutrient, and oxygen conditions are required in this step. The pH of the culture solution is generally adjusted using citric acid or sodium hydroxide. *Fusarium* utilizes nitrogen to produce amino acids, and then further synthesize proteins. Oxygen and glucose promote the aerobic respiration of fungus. After the fermentation process is complete, the broth is first heated in heat shock for 30–45 min above 68 °C, which prevents fungal growth and breaks down the ribosome, hydrolyzing the RNA to nucleotides to ensure that the nucleic acid content is below the WHO recommended intake of 2% *w*/*w* [36,37]. During this process, exogenetic nucleotidase is sometimes added to further break down the RNA. The resulting broth is then heated in a second step at 90 ℃, inactivating the fungus by altering its cellular structure and function, followed by homogenization and then centrifugation [38]. After centrifugation, the liquid supernatant and sediment are harvested. The sediment is a fungal protein biomass, which can be further processed into meat replacement products [39]. Subsequently, the sediment and supernatant are dried, and the drying step is essential for maintaining protein quality. At higher moisture levels, the availability of water for hydrating the reactants is sufficient to increase the reaction rate, which also increases the rate of diffusion of the reactant and might improve the solubility of the reactant [40]. Finally, the fungal protein biomass is further cooled to obtain fungal protein.

The main standard for improving fungal protein production is the selection of fungal strains with high protein contents and growth rates [41]. The ability to grow filamentous fungi is also regularly considered to be a selection standard for alternative protein production, as fungal hyphae are similar in size to muscle fibers and can imitate the texture of meat. Most fungi have complicated enzyme systems that allow them to make use of abundant substrates [8]. When endogenous enzymes are insufficient, different kinds of exogenous enzymes are often added to assist with fungal fermentation, thus producing products with different flavors. The cellulosic resources of industrial waste liquid, by-products from agricultural and sideline product processing, and all kinds of plant stalks, shells, sugar residue, and wood chips are usually used as raw materials for cultivating fungal biomass [42]. Gmoser et al. reintroduced by-products from food production, such as stale bread and beer waste grains, into the food production chain by adopting solid-state fermentation technology and utilizing the edible filamentous fungi *Neurospora intermedia* and *Rhizopusoryzae* for the biotransformation of by-products. As a result, the terminal fungal fermentation product has structural properties similar to commercial soy burgers, with the total protein content increased from 16.5% to 21.1% (dry weight) [43]. Different by-products have diverse chemical and physical properties, and various fungal strains differ in their ability to utilize these by-products. By cultivating strains on different materials, strains with a high yield, growth rate, and protein content are selected and optimized to achieve precise fermentation, so as to promote food fermentation efficiently and sustainably, and, ultimately, produce high-value foods [44]. 

## 3. Nutritional Value of Fungus Proteins

### 3.1. Flavor Quality of Fungus Proteins 

#### 3.1.1. Amino Acid Content and Composition

The nutritional value and quality of proteins from different sources are diverse; these are related to the structure of the protein and are measured by amino acid composition, the proportion of EAAs, the sensitivity to proteolysis during digestion, and the efficiency of protein processing [45]. Edible fungus proteins have a complete spectrum of EAAs, satisfy dietary requirements, and have economic superiorities compared with animal and plant sources. Protein nutritional value evaluation methods mainly include the protein content, chemical score (CS), amino acid score (AAS), essential amino acid index (EAAI), score of the ratio coefficient of the amino acid (SRCAA), biological value (BV), and nutritional index (NI), as well as other comprehensive evaluation methods. From a nutritional perspective, fungal proteins provide a range of valuable nutrients, including nine major EAAs, and their EAA composition accounts for 41% of the total protein, which is higher than most other familiarly used plant-based proteins [46]. Due to their relatively high protein quality, low saturated fat, and high fiber content, fungal proteins are essential for incorporating nutritious foods into the diet [47]. The EAA composition of fungal proteins is shown in Table 2; the EAAs in fungal proteins are comprehensive, and the proportions are relatively close to those in eggs. In addition, the bioavailability of all amino acids in fungal proteins is similar to that of milk, but superior to that of plant proteins [48]. Among the EAAs, lysine cannot be synthesized in the human body and must be supplied ex vivo, which is particularly important for infants and adolescents during their growth. The amino acid score after fungal protein digestibility correction is 0.996, which is close to milk, indicating that fungal proteins are high-quality proteins [49]. In addition, the composition and bioavailability of amino acids for fungal proteins might make them a potential source of protein to support protein metabolism in skeletal muscle [50].

#### 3.1.2. Flavor Characteristics of Fungal Proteins

Flavor control is an important feature of the end food products. In addition, taste and texture are important characteristics for evaluating foods. Among the five basic tastes, umami is preferred by most consumers, and umami is usually associated with meat-like, salty, and broth-like flavors [55]. The filamentous form of fungi can be used to give food products a meat-like texture, or be further processed to resemble meat [56]. The thickness of the texture of the prepared product can reach the length of the mycelium, which can be controlled by regulating the growth rate of the mycelium [57]. For fungus-based foods, fungal strains are a vital aspect of controlling the flavor of the final products. In addition, the choice of culture medium is a key factor that plays a role in delivering specific flavors to the fungus [58]. Depending on how fungal micro-organisms grow, the texture of fungus-derived foods can be controlled in a variety of ways. First, it can be changed by adding certain chemicals. Another factor that affects the texture of the product is the fungal form. For example, the oxygen content inside large fungal particles is extremely low, resulting in the internal autolysis of the particles; these can meet anaerobic conditions and produce hollow grain particles with a low biomass density. Fungus grown in a potato glucose broth may contain a different potato flavor, whereas fungus grown in a fruit-juice medium will retain the residual biomass components of some flavored juices. Kim et al. used sugarcane extract, NaNO_3_, and yeast extracts to culture Agaricus bisporus Suksung, obtaining meat analog products with good texture, hardness, elasticity, chewability, and umami characteristics. Scanning electron microscopy results showed that the product had a fibrous and oriented structure rather than a granular structure [59]. Al-Dalain explored the use of Shiitake mushroom in beef sausage formula, and the replacement treatment with 30% Shiitake mushroom had the best effect, increasing the total EAAs by 1.11 times. The total amount of non-EAAs was not affected by the replacement treatment, and the sample scored higher in sensory evaluation [60]. Patinho et al. evaluated the effect of Agaricus bisporus as a fat substitute for beef burgers. The study showed that adding mushroom could improve the moisture content and yield of hamburgers, and reduce the cooking loss of hamburgers, and the fat content was lowest when the content of Agaricus bisporus was 15%. The use of Agaricus bisporus also increases the oxidative stability of the product [61]. In the actual production process, bitter peptides will be produced in the process of protein enzymatic hydrolysis, which have an important impact on food flavor. The bitterness intensity of the bitter peptides is mainly determined by the type, structure, and arrangement order of the hydrophobic amino acids. Reducing the production of bitter compounds during enzymatic hydrolysis is a problem that needs to be solved. 

### 3.2. Nutritional Values of Fungus Proteins 

Food nutrition assessment should be based not only on the concentration of nutrients in food, but also on their bioavailability after digestion [62]. The body carries out physical, chemical, and enzymatic activities in the digestive tract, leading to the decomposition of the food matrix, and then releasing nutrients into the gastrointestinal tract and promoting their absorption; this process is called bioaccessibility. The process by which nutrients from food enter the serum through the intestinal epithelium is referred to as bioavailability [63]. Due to the porous nature of fungal protein cells, proteases in the gut can diffuse through the cell wall, which is a major factor in fungal proteolysis and bioaccessibility, rather than being due to mechanical/physical processing [23]. The correct selection of fungal species is essential to guide the manufacture of compounds with specific biological activity [64]. Edible fungi contain 10–63% protein (dry base), which contains sufficient essential amino acids (EAAs); is complementary to beans, vegetables, and dairy products; has a low fat content; and also contains B vitamins, a variety of minerals, and active polysaccharides [65]. Mushrooms do not contain vitamin D; instead, they contain high amounts of vitamin D precursor and can form vitamin D upon UV irradiation [66]. This is beneficial for calcium absorption in the body. 

Filamentous fungi are playing an increasingly important role in protein production through the modification of strains to produce multiple secreted proteins or particular target proteins, such as hydrophobic proteins or antimicrobial peptides, in highly specialized fungal hosts [67]. In addition, protein concentrates, enzymolysis, and peptides extracted from mushrooms are used for improving human health because of their antioxidant, anti-tumor, and antibacterial properties [68,69]. Basidiomycetes can produce ribonucleic-acid-hydrolyzed peptides homologous to ubiquitin sequences, as well as peptides and proteins with ribonuclease activity, which have been shown to have anti-proliferative activity against cancer cells [70]. Marson et al. found that a 15-kDa-molecular-weight cut-off membrane can effectively retain higher molecular weight compounds, and a fraction with bioactive potential, about 65% of the peptide less than 1 kg/moL, was prepared; this has high purity in terms of RNA and could be a choice for the pharmaceutical or food industry [71]. Ohata et al. fermented meat sauce from fermented pork flour, *Aspergillus*-fermented rice, and salt after 24 weeks. Fermented meat sauce can produce the antioxidant peptide Gln-Tyr-Pro through proteolysis, which has an antioxidant activity of more than 90% against OH free radicals [72]. Lam et al. isolated a new ribosome inactivation protein with a molecular weight of 20 kDa from the fruiting body of the mushroom *Lyophyllum shimeji*, which has antifungal activity against *Physalospora piricola* and *Coprinus comatus*. In addition, *Lyophyllum* antifungal protein with a molecular weight of 14 kDa was isolated, which has antifungal effects on *Physalospora piricola* and *Mycosphaerella arachidicola* [73]. Functional peptides with a variety of biological activities produced based on the decomposition of fungal proteins have great potential for development in the fields of food and medicine in the future, and may contribute to human health.

## 4. Bio-Functional Properties of Fungus Proteins 

### 4.1. Digestive Properties of Fungus Proteins

**Figure 3:**
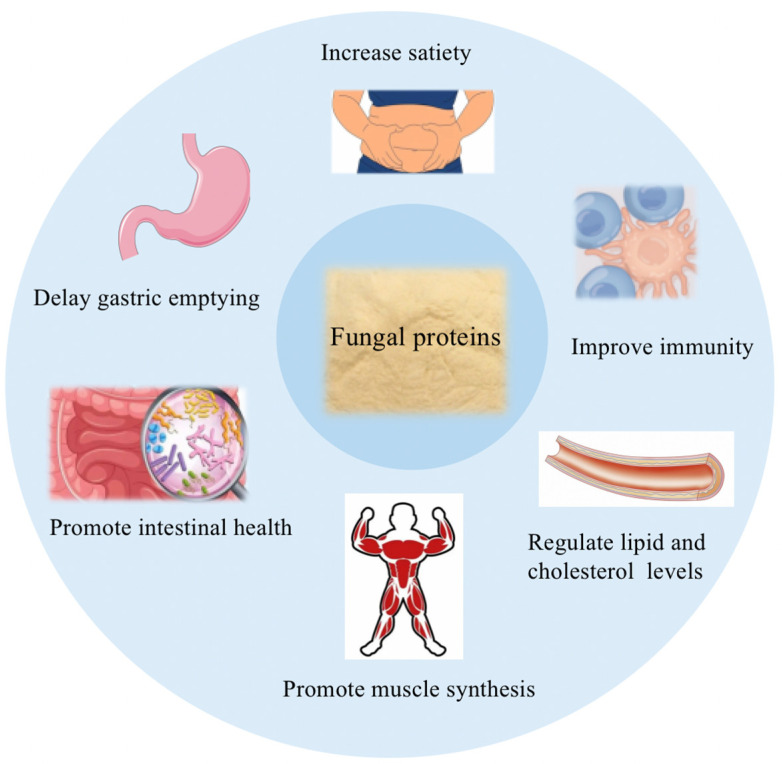
Health benefits of fungal proteins.

Figure 3 illustrates the health benefits of fungal proteins. Digestibility is an important indicator of protein bioavailability, and proteins with digestibility are usually associated with superior health; for example, faster protein digestion is linked to muscle anabolism. The products produced during protein digestion mostly depend on the structural attributes of foods, including the dissolvability of the protein, the availability of digestive enzymes, the protein sources of the food, and its processing attributes [74]. Fungi are easy to digest as the chitin wall provides a source of dietary fiber [75]. It has been found that the absorption kinetics of fungal proteins and the rising rate of EAAs in plasma are slower than those of milk, which is mainly due to the fiber content of the fungal protein cell wall affecting people’s digestive processes, such as delaying gastric emptying and facilitating the gastrointestinal tract absorption of nutrients [76]. Dunlop et al. investigated the impacts of fungal protein intake on acute postprandial hyperaminoacidemia and hyperinsulinemia in 12 healthy young men and found that the ingestion of fungal protein, a bioavailable source of insulin-stimulating dietary protein, led to slower but more persistent hyperinsulinemia and hyperaminoacidemia compared to milk [77]. Further, in a randomized controlled trial, researchers found that fungal protein reduced energy intake by 10% and insulin release (lowering insulin concentrations by 8%) in overweight people and regulated appetite compared with chicken [78]. In addition, fungal protein in the diet reduces energy intake in lean individuals. Clinical studies have shown no gastrointestinal discomfort or other adverse impacts during fungal protein interventions, and fungal-protein-based food substitutes are generally well-tolerated [79]. Colosimo et al. simulated in vitro digestion experiments showing that α-amylase hydrolyzed endocellular glycogen in fungal protein mycelia, and the concentration of glycogen amylase products in digestive fluid increased with digestion time. Moreover, the existence of fungal cell walls lowered the dynamics of reducing the sugar release during the digestive process compared to extracted glycogen [23]. Overall, fungal proteins have better digestive properties and are tolerated by patients with gastrointestinal function disorders.

### 4.2. Fungal Proteins Regulate Lipid and Cholesterol Levels

Fungal proteins can help maintain blood cholesterol levels within reasonable ranges, promote muscle growth, control blood sugar and insulin levels, and increase dietary satiety [46]. In addition, fungal proteins can reduce lipolysis because lipases can directly interfere with the fungal protein matrix. They can also bind bile salts. On the one hand, changes in pH have an effect on the cell wall structure of fungal proteins; on the other hand, trypsin activates protein hydrolysis. The viscosity of the fungal protein chyme was shown to be independent of the bile salt binding capacity during simulated glycemic index (GI) digestion [23]. As a soluble fiber in the fungal cell wall, β-glucan is associated with lower lipid levels. This result was demonstrated by Colosimo et al., who studied the release of β-glucan from fungal protein and white button mushroom and showed that uncooked fungal samples did not release β-glucan after simulated GI digestion, while, after stewing and digestion, β-glucan was released from the fungal protein matrix and its viscosity increased [80]. Ruxton et al. showed that the daily consumption of Quorn™ fungal protein for six weeks markedly reduced total cholesterol levels and low-density lipoprotein levels in humans [81]. By using Triton-X-100-induced hyperlipidemia in rat models, Thomas et al. demonstrated that fungal proteins significantly reduced blood lipid levels and significantly increased high-density lipoprotein levels in 100-, 200-, and 400-mg/kg-body-weight rats [82].

### 4.3. Fungal Proteins Improve Immunity

Fungal immunomodulatory protein (FIP), a micromolecular protein extracted from mushrooms, is a crucial bioactive ingredient of fungi with immunomodulatory activity, which can be used as an immunomodulator to prevent or restrain the occurrence of an exclusive reaction [83,84]. Bioactive proteins in mushrooms that may have immunomodulatory, anti-inflammatory, and anticancer activities have been widely studied for their immunopharmacological potential and are mainly divided into four categories based on their chemical properties: lectins, terpenes, proteins, and polysaccharides [85]. Structurally, FIPs consist of approximately 111–114 amino acid residues with an average molecular mass of about 12–15 kDa [86]. Most FIPs exist as homodimers, with each subunit composed of an N-terminal α-helical dimer and a C-terminal fibronectin III domain. FIPs have immunomodulatory and anti-inflammatory effects by suppressing the overproduction of Th2 cytokines that are common in allergic reactions [87]. One group of FIPs found in fungi, a subset of type-5 FIPs, has been most extensively studied for its hemagglutination, immunomodulatory, and anticancer properties [17]. In addition, FIPs might have anticancer activity, such as the fungal immunomodulatory protein *G. tsugae*, which has been shown to inhibit the growth of A549 lung cancer cells in vitro through restraining telomerase activity [88]. Further, fungal immunomodulatory proteins extracted from *Ganoderma microsporum* have anti-inflammatory and neuroprotective potential in primary cultured rodent models in vitro [86]. Therefore, mushrooms are used more in the food industry as a kind of fungus with the same origin as medicinal and edible products. At present, there are few studies on this aspect, and more studies are needed to prove that fungal proteins could enhance immunity.

### 4.4. Fungal Proteins Promote Gut Health

An imbalance of the gut microbiota can alter intestinal permeability, damage the integrity of the intestinal barrier, and affect the human metabolism and immune response. The pro-inflammatory state caused by changes in the intestinal flora balance can lead to many diseases, ranging from gastrointestinal diseases to immune and neuropsychiatric diseases, so maintaining intestinal health is crucial to human health [89]. In industrial production, mycoprotein is a meat substitute produced primarily using *Fusarium venenatum*, which is high in protein and dietary fiber. The fibrous portion of the fungal protein comprises 1,3/1,6 β-glucan, chitin, and a small amount of mannoprotein. A blind, randomized, cross-diet intervention trial of 20 healthy adult men showed that substituting red and overprocessed meat with *Fusarium*-containing meat substitutes (fungal proteins) reduced fecal genotoxicity and genotoxin excretion, and enhanced the abundance of health-friendly microbiome genera (*Lactobacilli*, *Roseburia*, and *Akkermansia*) in the gut [90]. Diling et al. isolated a single-band protein from *Hericium erinaceus* that regulated the composition and metabolism of cyclophosphamide-induced gut microbiota in mice, activated T-cell proliferation and differentiation, and played a role in an antibiotic overdose inflammatory bowel disease model [91]. Thus, fungal proteins might be a beneficial alternative to meat in terms of gut health and colorectal cancer prevention. 

## 5. Application of Fungal Proteins 

### 5.1. Application of Fungal Proteins in Foods

The branching properties of fungal mycelia are similar to the organization of muscle fibers and contribute to the development of meat-like structures. At present, fungal-protein-based food products mainly include meatless chicken patties, breakfast patties, sausages, and burgers, and dairy-free cream cheese. Gamarra-Castillo et al. used fungal proteins to develop burger patties that are rich in protein and fiber. In addition, an in-depth and long-term shelf-life analysis of fungal-protein-related products, combined with a flavor analysis and nutritional value evaluation, were carried out to evaluate the popularity and acceptance of fungal protein products in the market [92,93]. Typical foods prepared using fungal proteins are listed in Table 3. Edible fungi are part of the human food system and are considered a vital part of a healthy human diet; they are usually consumed in the form of mushrooms or as components of food products, mostly belonging to basidiomycetes. Edible fungi are collected or grown all over the world, such as *Bisporus mushroom*, *Pleurotus pleurotus*, *Shiitake mushroom*, *Flammulina velutipes*, and *Volvariella volvacea*, with substantial growth in cultivation [94]. As a sustainable mode of production, mushrooms are often grown commercially using agricultural residues or by-products, and can convert these wastes into a valuable food source for human consumption.

As a sustainable source of dietary proteins, fungal proteins have been declared as safe products by the US Food and Drug Administration (FDA) [103,104] and could be included in the daily diet. Fungi-fermented foods are an integral part of the diet in many countries, and are divided into legume or cereal high-protein meat substitutes such as tempeh and Oncom, and salted sauces such as tofu and miso [105]. *Penicillium roquefortii* and *Penicillium camembertii* are used to produce blue and soft ripe cheeses, respectively. *Monascus* is used to produce monascus rice; *Aspergillus oryzae* can be used to ferment soybeans to produce ham, miso, and tofu. Filamentous fungi can be further processed to produce fungal proteins [106]. Since different strains can produce different specialized metabolites, they are often key components of nutritional supplements. In addition to well-established methods of producing fungal proteins that are already available, the health benefits are also being proved. Thus, fungal-protein-based foods can partially substitute animal-based protein foods. 

### 5.2. Application of Fungal Proteins in Space Agriculture

Fungi are both producers of products and decomposers and recyclers of waste, and may become important drivers of a circular economy in sealed environments [107]. The International Space Station is a closed system. The modern biotechnology of fungi (that is, the controlled culture in bioreactors) and industrial production of fungal proteins require advanced mechanical equipment. Filamentous fungi are well-suited for the sustainable production of commodities on Earth and in space. In particular, *Aspergillus niger* can produce different kinds of products due to its undemanding survival conditions, high reproductive efficiency, and metabolic diversity. Thus, as a necessity of space travel in the 21st century and beyond, the filamentous fungus *Aspergillus niger* could become an important raw material for astronauts to independently produce food, enzymes, and antibiotics during space travel [108]. However, due to the difference between the space environment and the Earth, the production of fungal proteins in the space environment needs to include a consideration of the aspects of fungal culture, reproduction biology, life support systems under weightlessness, and so on [109].

## 6. Challenges and Future Trends

With the increasing attention on fungal proteins, one of the ways to develop high-value edible fungal products is to prepare edible fungal proteins and separate them from other components. The growth of filamentous fungi is cyclical, and mycelium autolysis occurs after aging, so vigorous mycelia or fruiting bodies should be selected when preparing proteins. At present, the application method for edible fungal protein is mainly to add it directly. There are few reports on deep processing as an important intermediate link between development and utilization compared with plant proteins, including the processing characteristics and production technology. Edible fungi contain more fibers and soluble polysaccharides, and their toughness and viscosity are different from those of other materials [65]. Thus, it is necessary to carry out targeted research and establish further processing technology with strong applicability. The consumption of fungal protein can alleviate the protein supply gap; provide consumers with healthy, low-fat protein with diversified flavors; and reduce the pressure on the ecological environment. More commonly, consumers have a low acceptance of highly processed products comprising fungal protein. On the one hand, compared with traditional livestock and poultry meat products, the imitation meat processed using fungal protein has a lower fat content and a less oily aroma. On the other hand, fungal protein lacks the hemoglobin contained in meat, so it lacks the characteristic “meaty fishy” taste of meat [110]. It is essential to further optimize the appearance of edible fungal protein products, enhance the taste, and improve consumers’ awareness and acceptance of new protein products. The species of edible fungi used for protein production determine the production cycle, cost, and product quality. Breeding is an important way to obtain high-yield and high-quality edible fungal varieties, which is closely related to the development of the fungal protein industry. The main edible fungi sold in the market are basidiomycetes such as *Shiitoki mushroom*, *Pleurotus edodes*, and *Bisporus mushroom*, which have relatively good consumer acceptance. Therefore, the screening and molecular breeding of *Basidiomycota* can be further carried out to obtain strains exhibiting high protein production. In 2001, the U.S. Food and Drug Administration declared that fungal proteins are safe to use in foods other than poultry and meat. Furey et al. extracted Fy Protein™ from the fermented *Fusarium* strain flavolapis, and developed it as an alternative to animal-based protein foods such as meat and dairy products, demonstrating its lower toxicology, genotoxicity, pathogenicity, and sensitization in rats [111]. Fungal protein intake is associated with reduced total cholesterol levels, especially in patients with hyperlipidemia. In addition, fungal proteins can induce muscle protein synthesis [24]. Although fungal proteins have some potential benefits for the human body, questions regarding the sensory and health experience of fungal proteins are still under investigation [112]. Pathogens such as *Escherichia coli*, *Salmonella*, *Staphylococcus aureus*, and *Listeria* are sometimes detected in fungal fermented foods, which may be a potential health threat to consumers due to the inadequate sanitary conditions during the manufacturing process and the lack of safety control standards [113]. 

## 7. Conclusions and Prospects

As a wide variety of tiny organisms on Earth, fungi play an important role in people’s lives, from enzyme preparations and the environment to medical and food industries. Fungal protein prepared from fungi has high nutritional value, which is manifested in its high protein content, richness in vitamins, low fat content, and absence of cholesterol. In addition, fungal proteins are rich in amino acids and fatty acids. In industrial production, the selection and cultivation of strains, fermentation, and other preparation processes affect the quality of fungal proteins, including their flavor and bioavailability. In addition to their nutritional value, fungal proteins have a variety of functional properties, including regulating lipid and cholesterol levels, increasing satiety, promoting digestion, improving immunity, and facilitating intestinal health. Fungal proteins are increasingly used in the food industry, particularly as meat substitutes. Many other foods have also been derived from fungi, such as hamburgers, cheeses, sauces, and so on. 

With global protein resources strained, producing large quantities of tasty, nutritious, safe, and sustainable protein is an urgent matter. Fungal-protein-based foods are increasingly available, but the overall acceptance of fungal proteins is still low. Therefore, the flavor of fungal protein products needs to be further improved to meet the needs of consumers. Moreover, it is necessary to further explore the high-value and efficient preparation and processing technology of edible fungal proteins, realize multi-dimensional high-value utilization, and promote the iterative development of edible fungal protein products and production strains. The production of fungal protein also needs to rely on the traditional fermentation process, so it is necessary to accelerate the fermentation process, increase the product yield, and, ultimately, improve the quality, safety, nutrition, and flavor of fungal-protein-based foods. In addition, further research is needed to investigate the effects of fungal protein consumption over longer periods of time and in different populations, such as healthy individuals and metabolically impaired individuals.

## Figures and Tables

**Table 1:** The composition of fungal proteins (taking Quorn mycoprotein as an example, produced using *Fusarium venenatum*) (https://www.quornnutrition.com/importance-of-micronutrients, accessed on 16 June 2023).

Nutrients	Mycoprotein (Wet Weight, %)
Protein	11.0
Carbohydrate	3.0
Lipids	2.90
Dietary fiber	6.0
Vitamin B6	0.0001
Vitamin B9 (folate)	0.000114
Riboflavin	0.00026
Choline	0.00018
Sodium	0.005
Manganese	0.0049
Magnesium	0.049
Calcium	0.048
Phosphorus	0.29
Potassium	0.071
Zinc	0.0076
Energy (kcals/100 g)	85

**Table 2:** Composition of amino acids in fungal proteins and other foods [51,52,53,54].

Amino Acids	Amino Acid Content (g/100 g Protein)			
	Mycoprotein	Milk Protein	Egg White	Soy Protein
His	3.50	2.37	2.10	2.40
Ile	5.70	4.57	4.40	4.00
Leu	8.60	9.66	7.30	7.00
Lys	8.30	7.78	6.10	5.50
Met	2.10	2.40	3.20	1.10
Phe	4.90	4.71	5.10	4.60
Thr	1.80	4.18	4.00	3.70
Val	6.20	5.33	5.80	4.10
Cys	0.80	0.90	2.50	1.10
Arg	7.30	3.50	5.00	6.90
Tyr	4.00	4.90	3.90	3.50
Asp	10.30	7.50	9.30	10.00
Ser	5.10	5.20	6.00	5.10

**Table 3:** Typical foods prepared using fungal proteins.

Fungi	Fungal-Protein-Based Products		Source	Reference
*Mold* (*Aspergillus oryzae*)	Miso	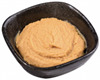	Soybean.	[95]
*Aspergillus oryzae*	Soy sauce	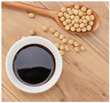	Soybean.	[96]
*Rhizopus oligosporus*	Tempeh	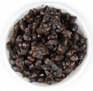	*Rhizopus oligosporus* is subjected to six-day solid-state fermentation in broad beans.	[97]
*Penicillium roquefortii Penicillium camembertii*	Blue cheeses	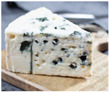	The source of mycelia can be milk, the natural environment, or starter cultures added to milk.	[98]
*Fusarium*	Dairy-free cream cheese	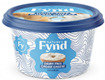	Fungal protein Fy™.	[47]
*Aspergillus oryzae*	Burgers—meat substitute	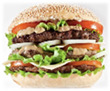	*Aspergillus oryzae* was cultivated using maltodextrin as a carbon source, supplemented with beet extract, carboxymethyl cellulose, and quinoa flour.	[92]
*Fusarium*	Meatless breakfast patties	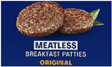	Fungal protein Fy™ with all nine essential amino acids.	[99]
*Fusarium venenatum*	Animal meat substitutes	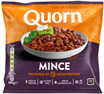	Fungal protein, egg white, and wheat flour. Rich in protein and fiber; soy-free.	[100]
*Pleurotus sapidus*	Vegetarian sausage	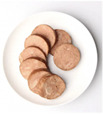	*Pleurotus sapidus* mycelium grown on isomaltulose molasses or apple pomace.	[101]
*Mushroom mycelium*	Plant-based bacon	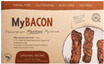	*Mushroom mycelium*, salt, coconut oil, sugar, natural spices, concentrated beet juice.	[102]

## Data Availability

The data used to support the findings of this study can be made available by the corresponding author upon request.
